# CART treatment improves memory and synaptic structure in APP/PS1 mice

**DOI:** 10.1038/srep10224

**Published:** 2015-05-11

**Authors:** Jia-li Jin, Anthony K.F. Liou, Yejie Shi, Kai-lin Yin, Ling Chen, Ling-ling Li, Xiao-lei Zhu, Lai Qian, Rong Yang, Jun Chen, Yun Xu

**Affiliations:** 1 Department of Neurology, Affiliated Drum Tower Hospital of Nanjing University Medical School, Nanjing, China; 2 Department of Neurology, University of Pittsburgh, Pittsburgh, PA, USA; 3 The State Key Laboratory of Pharmaceutical Biotechnology, Nanjing University, Nanjing, China; 4 Department of Physiology, Nanjing Medical University, Nanjing, China

## Abstract

Major characteristics of Alzheimer’s disease (AD) include deposits of β-amyloid (Aβ) peptide in the brain, loss of synapses, and cognitive dysfunction. Cocaine- and amphetamine-regulated transcript (CART) has recently been reported to attenuate Aβ-induced toxicity. In this study, CART localization in APP/PS1 mice was characterized and the protective effects of exogenous CART treatment were examined. Compared to age-matched wild type mice, 8-month-old APP/PS1 mice had significantly greater CART immunoreactivity in the hippocampus and cortex. A strikingly similar pattern of Aβ plaque-associated CART immunoreactivity was observed in the cortex of AD cases. Treatment of APP/PS1 mice with exogenous CART ameliorated memory deficits; this effect was associated with improvements in synaptic ultrastructure and long-term potentiation, but not a reduction of the Aβ plaques. Exogenous CART treatment in APP/PS1 mice prevented depolarization of the mitochondrial membrane and stimulated mitochondrial complex I and II activities, resulting in an increase in ATP levels. CART treatment of APP/PS1 mice also reduced reactive oxygen species and 4-hydroxynonenal, and mitigated oxidative DNA damage. In summary, CART treatment reduced multiple neuropathological measures and improved memory in APP/PS1 mice, and may therefore be a promising and novel therapy for AD.

Alzheimer’s disease (AD) is the most prevalent neurodegenerative disease associated with dementia, with more than 26 million cases worldwide in 2006 and an expected 106 million cases by 2050^1^. Little is known about the etiology of AD and the identification of a cure has remained elusive. Recent studies have shown that cocaine- and amphetamine-regulated transcripts (CART) can mitigate cell death and neurological impairments induced by ischemia^2^,^3^,^4^. Thus, the present study tests whether CART shows promise as a novel therapeutic candidate for the treatment of AD.

CART is an anorexigenic neuropeptide produced by the pituitary gland, adrenal gland, and pancreas, and is widely distributed throughout the central and peripheral nervous systems^5^. CART regulates food consumption, body weight, stress control, reward, and pain transmission. Recently, we reported that icariin-mediated protection against amyloid-β (Aβ)-induced neuronal death was modulated by upregulation of CART^6^. Furthermore, CART treatment attenuated neuronal loss in the substantia nigra and mitigated behavioral deficits in the MPTP model of Parkinson’s disease^7^,^8^. These studies suggest that this neuropeptide may alleviate the pathology associated with neurodegenerative diseases.

The mechanisms underlying the neuroprotective effects of CART are still under investigation. CART treatment is known to promote the *in vitro* survival of cultured hippocampal neurons by upregulating brain-derived neurotrophic factor^9^. In an ischemic injury model, protection by estrogen was mediated by an increase in endogenous CART and activation of the ERK signal transduction cascade^3^,^4^. Similarly, CART-mediated ERK signaling mitigated cortical neuronal demise in response to Aβ toxicity^6^. Further mechanistic examinations have indicated that ischemia-induced suppression of CART expression was modulated by the transcriptional repressor known as neuron-restrictive silencer element^10^. In addition, it has been proposed that attenuation of dopaminergic neuronal cell loss after CART treatment in MPTP-treated mice could be attributed to the anti-oxidative properties of CART^7^.

In the present study, the protective properties of CART peptide in *in vitro* and *in vivo* models of AD are characterized. It is demonstrated, using immunostaining, that CART expression is naturally elevated in the brains of APP/PS1 transgenic mice and in AD patients in the Aβ plaques, perhaps as a compensatory defense mechanism. It is also observed that CART treatment preserves synapse and dendritic structure in cellular and animal models of AD. Results suggest that the mechanisms underlying CART-mediated neuroprotection include attenuation of oxidative damage and improvement of mitochondrial function. These findings support the notion that CART-based therapies may be beneficial in AD patients.

## Results

### Regional distribution of CART in the brains of APP/PS1 transgenic mice and AD patients

**Figure 1:**
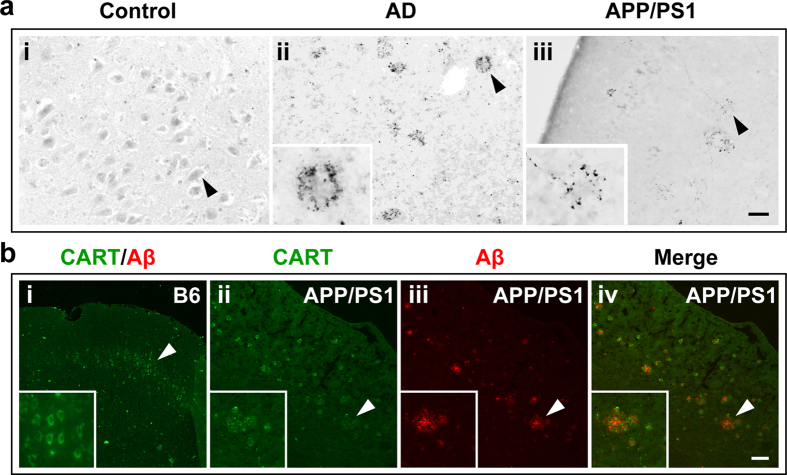
CART immunoreactivity is prominent in cortical Aβ plaques in AD. (**a**) CART immunostaining is distributed uniformly in cell bodies of cortical neuron-like cells in a control non-AD patient (**i**) and concentrated around the cores of Aβ plaque-like structures in AD brain (representative of three non-AD and three AD brains) (**ii**) and in 10-month-old APP/PS1 transgenic mice (**iii**). Scale bar =100 μm. (**b**) Similar staining patterns for CART (green) and Aβ (red) are seen in 8-month-old APP/PS1 mice, where the CART signal was most prominent in the regions surrounding the plaques but not at the core of the plaques (indicated by white arrows). Scale bar =100 μm. The images are representatives of 3 mice per phenotype with similar results.

In an effort to test whether CART expression is altered in AD brain, cortical tissues from three AD patients and three age-matched non-demented individuals were subjected to immunohistochemistry for CART. In controls, weak immunoreactivity for CART was barely detectable in the cortex (Fig.1a, arrow within panel i). However, in the cortex of AD patients, there was increased CART immunoreactivity with punctuate distribution surrounding structures that were speculative of Aβ plaques (Fig. 1a inset and arrow within panel ii). Similarly, increased CART immunoreactivity pattern was also found in the cortex of 10-month-old APP/PS1 mice (Fig. 1a inset and arrow within panel iii). To determine the possible association between elevated CART immunoreactivity and Aβ plaques, double staining of CART and Aβ was performed in 8-month-old APP/PS1 mice. As shown in Fig. 1b, punctuate CART staining was found in clusters that surrounded and/or were within Aβ plaques.

**Figure 2:**
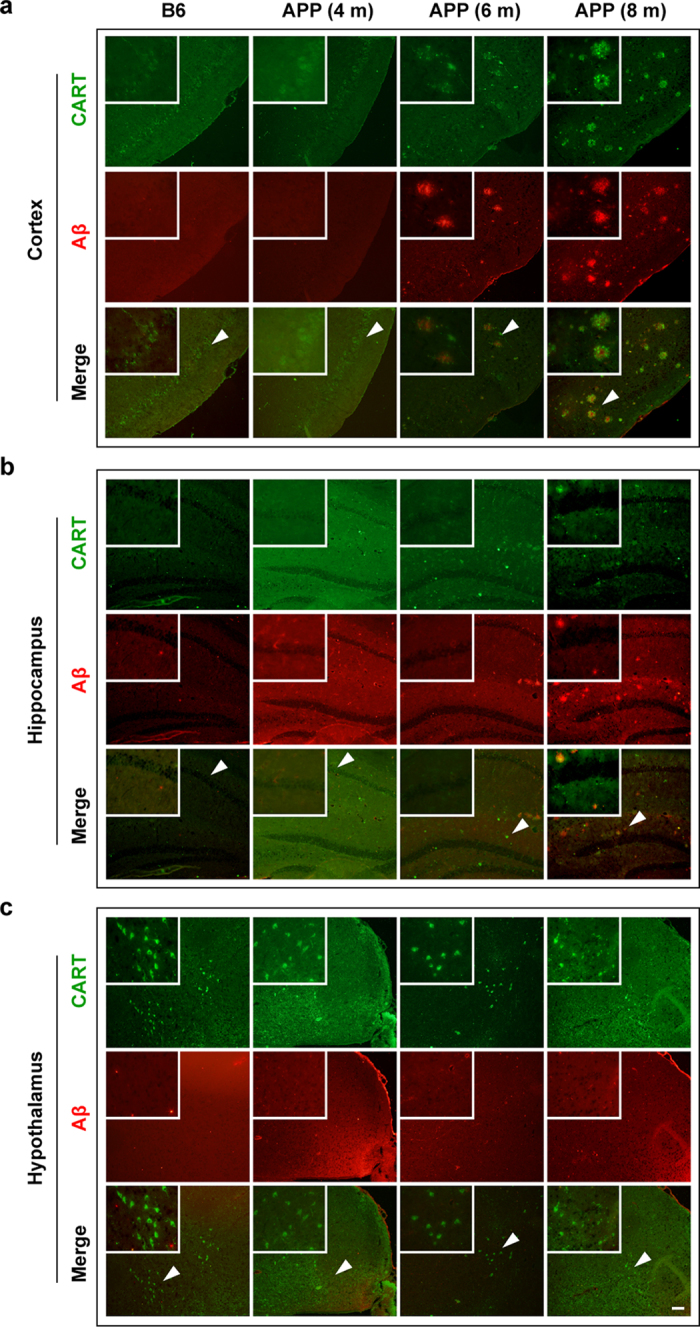
CART and Aβ expression and distribution in the cortex (a), hippocampus (b), and hypothalamus (c) of APP/PS1 transgenic mice and B6 control mice. CART (green) immunofluorescence is prominent in cell bodies and neurites in B6 control mice, and lower in 4-month-old APP/PS1 transgenic mice in the absence of Aβ plaques. In 6- and 8-month-old APP/PS1 mice, where Aβ plaques (red fluorescence) had emerged in the cortex and hippocampus, CART was localized in Aβ plaques. CART immunoreactivity was also prominent in cell bodies in the hypothalamus. Arrows indicate the location of CART immunoreactive structures shown in the insets. Scale bar = 100 μm. Images are representatives of 3 mice per group with similar results.

To further characterize the temporal profile of CART immunoreactivity and its association with Aβ plaque formation, a series of double-label immunostaining for CART and Aβ was performed in APP/PS1 mice at 4 (prior to the onset of β-amyloid plaques), 6 and 8 (after β-amyloid plaque formation) months old and in age-matched control C57BL/6 (B6) mice. In the cortex and hypothalamus of both APP/PS1 mice at 4 months of age and of control B6 mice, CART immunoreactivity exhibited a similar distribution. Regardless of genetic profile, CART expression was more prominent in the hypothalamus than in the cortex and hippocampus, and was mainly localized in neuron-like cells (Fig. 2a,b and c, top panels). By contrast, in 6- and 8-month-old APP/PS1 mice, Aβ plaques were widely detectable in the cortex and hippocampus, and increased clusters of CART immunoreactivity was found to surround and/or to be within β-amyloid plaques (Fig. 2a,b and c, bottom panels).

### CART expression in the brains of APP/PS1 transgenic mice

**Figure 3:**
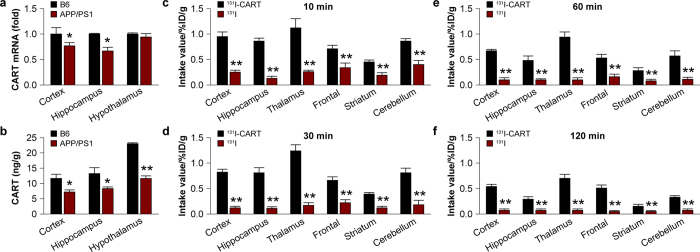
Expression of endogenous CART in 8-month-old APP/PS1 mice and the capacity of exogenous CART to cross the blood-brain-barrier. (**a**) CART mRNA levels in the cortex, hippocampus and hypothalamus in 8-month-old APP/PS1 mice and B6 control mice. All values are expressed as mean ± SEM, n = 6 mice/group, *p < 0.05 versus B6 control mice. (**b**) CART protein levels were measured by ELISA in the cortex, hippocampus and hypothalamus in 8-month-old APP/PS1 mice and B6 control mice. All values are expressed as mean ± SEM, n = 6 mice/group, *p < 0.05; **p < 0.01 versus B6 control mice. The capacity of CART to cross the blood-brain barrier after intravenous injection of pre-radiolabelled ^131^I CART peptide was compared to a control ^131^I solution. The retention of radiolabelled CART in various brain regions is shown at 10 minutes (**c**), 30 minutes (**d**), 60 minutes (**e**), and 120 minutes (**f**) after injection. The ^131^I solution exhibited no retention 120 minutes after injection. On the other hand, CART peptide exhibited high retention in the thalamus, cortex and hippocampus after influx into the brain. All values are expressed as mean ± SEM, n = 6 mice/group, **p < 0.01 versus ^131^I solution.

CART mRNA and protein levels in the cortex, hippocampus, and hypothalamus of 8-month-old APP/PS1 transgenic and age-matched B6 mice were next analyzed. In APP/PS1 mice, CART mRNA levels were lower by 23.2% and 33.5% (Fig. 3a) and CART protein levels were decreased by 13.5% and 20.9% in the cortex and hippocampus, compared to B6 control mice as measured by ELISA (Fig. 3b). In the hypothalamus, APP/PS1 mice had similar CART mRNA levels but lower protein levels compared to control mice (Fig. 3a,b).

### Exogenous CART attenuates memory deficits in APP/PS1 mice

Next, the effects of exogenous CART in 8-month-old APP/PS1 mice were assessed. The delivery method of CART peptide into the mice was dependent on its inherent capacity to cross the blood-brain barrier. Using radiolabeled CART peptide (^131^I-CART) and iodine-131 solution as a positive control, the radiolabeled peptide or solution was injected intravenously followed by determination of distribution in various brain regions of B6 mice at 10, 30, 60, and 120 minutes post-injection. As expected, the radiolabeled iodine solution crossed the blood-brain barrier and distributed in all parts of the brain with low retention time (Fig. 3c-f). At 120 minutes, more than 70% of the iodine solution had exited the brain.

CART crossed the blood-brain barrier with a distribution pattern similar to the iodine solution at 10 minutes post-injection (Fig. 3c). However, CART exhibited a much higher retention time than the iodine solution. After 120 minutes, the CART peptide was preferentially retained in the thalamus and cortex. Intravenous injection was subsequently selected over stereotaxic injection as the delivery method to minimize surgical injury. In the determination of the minimum effective dosage, the effects of CART on 8-month-old APP/PS1 and age-matched B6 mice via injection at 0.5, 1.0 and 2.0 μg/kg for 30 days with saline as vehicle were investigated. At these doses, there was no difference in average body weight and movement speed of the transgenic mice before and after CART injections (data not shown). As all three doses of CART ameliorated behavioral deficits in APP/PS1 mice (data not shown), the lowest dose (0.5 μg/kg) was used in all subsequent experiments.

**Figure 4:**
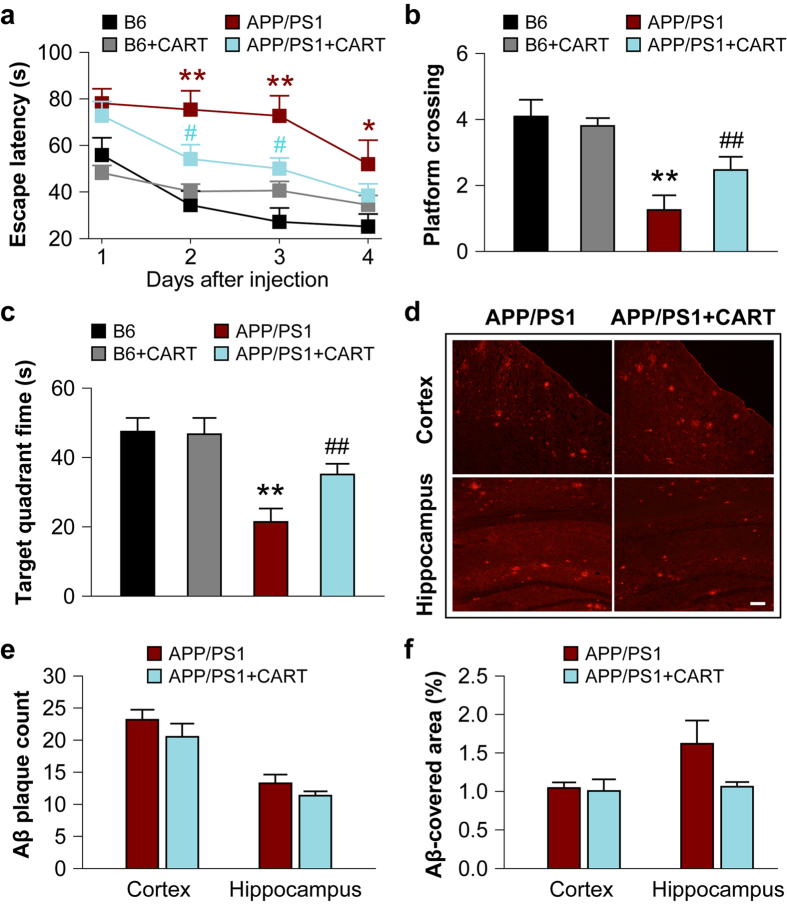
Effects of exogenous CART treatment on memory impairments and Aβ plaque load in APP/PS1 mice. (**a**) CART treatment of APP/PS1 transgenic mice significantly lowered the latency to escape a submerged platform in the Morris water maze test at 8 months of age. All data are expressed as mean ± SEM, n = 15 mice/group, *p < 0.05, **p < 0.01 versus B6 control mice. ^#^*p *< 0.05 versus APP/PS1 transgenic mice without CART treatment. CART-treated APP/PS1 mice also made higher numbers of passes over the submerged platform (**b**) and spent more time in the target quadrant (**c**) compared to APP/PS1 mice without CART treatment. All data are expressed as mean ± SEM, n = 15 mice/group, **p < 0.01 versus B6 control mice, ^##^*p *< 0.01 versus APP/PS1 transgenic mice without CART treatment. (**d**) Aβ-immunoreactive plaques in 8-month-old APP/PS1 transgenic mice in the absence or presence of CART, Scale bar: 100 μm. Counts of total Aβ plaque numbers (**e**) and Aβ plaque load (**f**) in the cortex and hippocampus of 8-month-old APP/PS1 mice (6–12 sections/mouse). All values are expressed as mean ± SEM, n = 3 mice/group.

The Morris water maze (MWM) test was administered before CART treatment (to confirm behavioral deficits occurring in APP/PS1 mice) and 72 hours after the final CART injection. The MWM tests demonstrated that escape latencies (time needed to locate the platform) were comparable in APP/PS1 mice, with and without CART peptides, but were longer than in B6 control mice with and without CART injections during the first trial (p < 0.05, Fig. 4a). However, APP/PS1 mice treated with CART exhibited significantly lower escape latencies (p < 0.01) than APP/PS1 mice without CART in three subsequent consecutive trials (Fig. 4a). These results indicated that CART treatment improved cognition and memory in APP/PS1 mice. Upon submerging the platform, APP/PS1 mice with CART treatment crossed the area of the platform more frequently (2.46 ± 0.40 vs. 1.25 ± 0.45) and spent more time in the target quadrant (35.08 ± 3.09% vs. 21.39 ± 3.87%) (Fig. 4b, c).

### CART dose not reduce Aβ plaques in the brains of APP/PS1 mice

Aβ plaque numbers in the cortex and hippocampus of APP/PS1 mice were then quantified (Fig. 4d-f), which indicated that there was no significant difference in Aβ plaque formation after CART treatment (p > 0.05).

### CART preserves neurite and synapse structure in APP/PS1 mice

**Figure 5:**
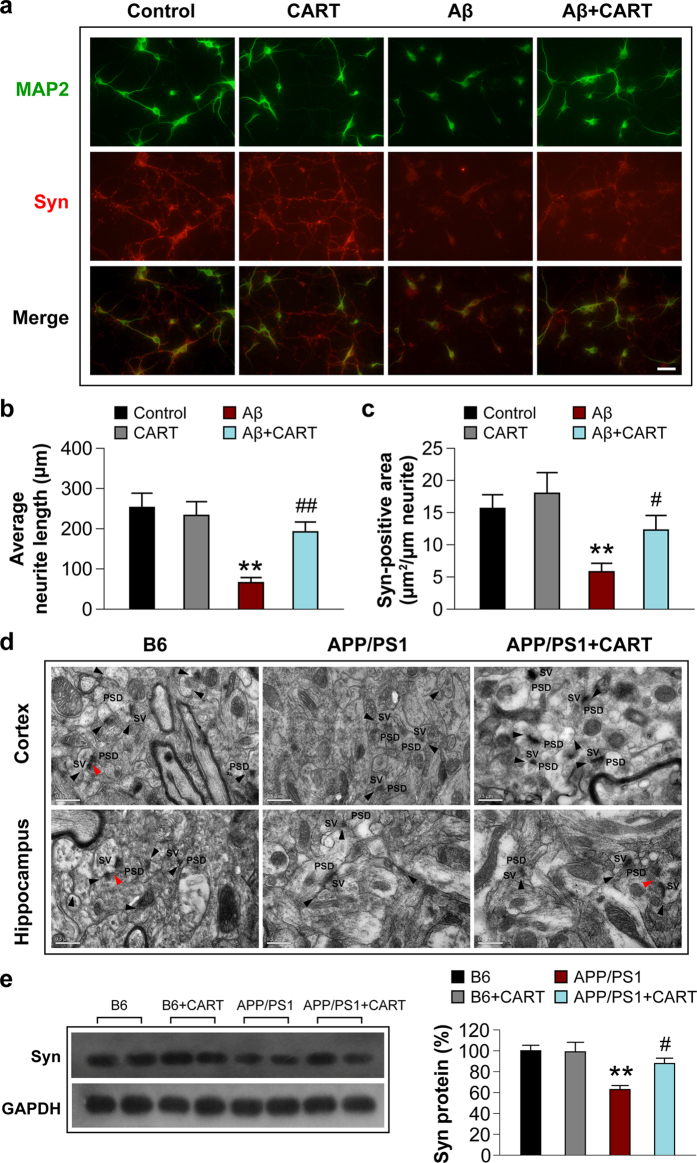
CART treatment protects neurites against Aβ toxicity in cultured cortical neurons and preserves synaptic structures in APP/PS1 mouse brain. (**a**) CART treatment prevents loss of neurite structures (MAP2, green) and enhances synaptophysin (red) expression in cultured cortical neurons. Scale bar =40 μm. CART treatment increased the average neurite length compared to no treatment (**b**), and increased synaptophysin-positive areas within neurites (**c**). All values are mean ± SEM of data from at least 45 randomly selected images for each assay, based on 3 independent experiments using different primary culture preparations. **p < 0.01 versus B6 control, ^#^*p *< 0.05 versus cortical neurons treated with Aβ_1-42_ only. (**d**) CART treatment preserves synaptic ultrastructure in the cortex and hippocampus of APP/PS1 mice (8 months), as visualized by electron microscopy. Electron micrographs illustrate synaptic ultrastructure in the cortex (upper three panels) and hippocampus (lower three panels) of B6 control mice, untreated APP/PS1 mice, and APP/PS1 mice with CART treatment. The black arrows indicate synaptic clefts; the red arrows indicate perforated synapses. PSD, postsynaptic density; SV, synaptic vesicles. Scale bar =0.5 μm. (**e**) CART treatment elevated synaptophysin levels in the hippocampus, as determined by Western blot analysis followed by quantification. The blot shown here is a representative of 3 independent experiments. Blot images were cropped for comparison. All values are expressed as mean ±SEM of the band intensity of synaptophysin normalized to GAPDH from 3 independent experiments, **p < 0.01 versus B6 control mice, ^#^*p *< 0.05 versus APP/PS1 mice without CART treatment.

Neurite structures were examined using MAP-2 immunostaining and synapses were examined using synaptophysin immunostaining. In Aβ_1-42_-treated cortical neurons, an amount lower than or comparable to previous studies^11^,^12^,^13^ for 24 hours elicited significant loss of neurite structures and synaptophysin protein. However, pretreatment with CART attenuated neurite degeneration and increased synaptophysin protein levels (Fig. 5a). Neurite length was increased from 65.7 ± 12.7 to 191.9 ± 24.4 μm/cell with CART pretreatment (Fig. 5b). In addition, the synaptophysin-positive area within the neurite was about 2 times higher, increasing from 5.8 ± 1.3 to 12.3 ± 2.3 μm^2^/μm neurite in the presence of CART (Fig. 5c).

Next, the effect of CART on synapses in the cortex and hippocampus in APP/PS1 mice was evaluated by transmission electron microscopy. In the cortex and CA1 area of the hippocampus of 8-month-old APP/PS1 mice, the number of synapses was much lower than that in B6 control mice. By contrast, B6 mice exhibited more synaptic vesicles in the presynaptic compartment and showed the typical asymmetric appearance of Gray’s type I synapses (Red arrow, Fig. 5d). Synaptic ultrastructure also appeared severely compromised in both regions in APP/PS1 mice. However, degeneration of synaptic architecture in cortex and hippocampus was partially reversed by CART treatment (Fig. 5d). This increase in synapses was accompanied by a 39.5% increase in synaptophysin protein levels in the hippocampus of APP/PS1 mice treated with CART (Fig. 5e).

**Figure 6:**
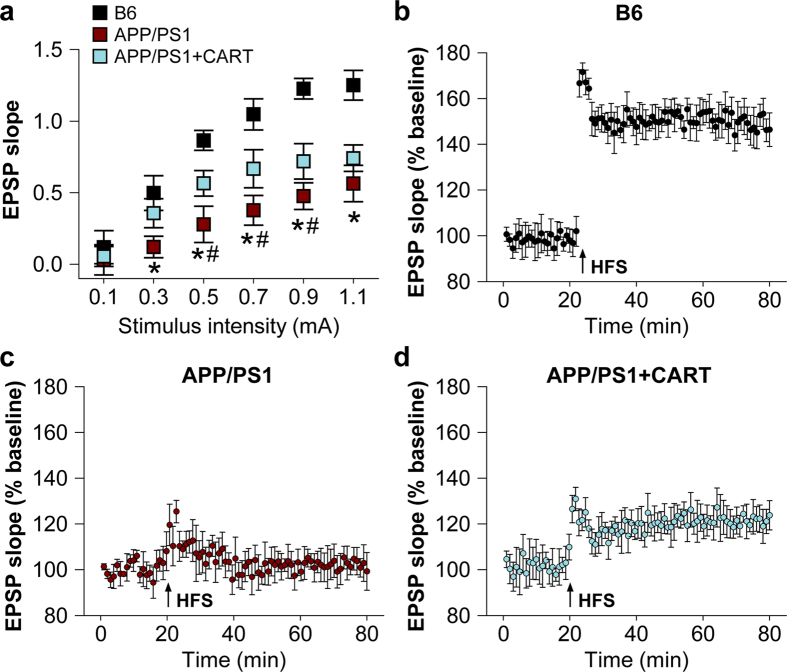
CART treatment facilitated functional recovery of synapses in APP/PS1 mice. Electrophysiological determination of deficits in Schaffer collateral-CA1 synaptic transmission and the induction of long-term potentiation. (**a**) A plot showing input/output (I/O) curves in response to stimulus (0.1-1.1 mA) at the hippocampal CA1 region of B6 control mice, APP/PS1 mice, and APP/PS1 mice receiving CART treatment (8 months old). The I/O curve was measured by averaging the slope of EPSPs against stimulus intensity at 0.1-1.1 mA. The slope of the regression line for the I/O curve in APP/PS1 mice was markedly reduced. With CART treatment, the reduction in I/O curve slope was significantly improved for APP/PS1 mice. All values are expressed as mean ± SEM from 6 mice in each group. *p < 0.05 versus B6 control mice, ^#^*p *< 0.05 versus APP/PS1+CART. The capacity to establish long-term potentiation (LTP) from high-frequency stimulus (HFS, 100 pulses at 100 Hz) with the same pre-HFS intensity at hippocampal CA1 region of B6 control mice (**b**), APP/PS1 transgenic mice (**c**) and APP/PS1 mice with CART treatment (**d**).

Whether CART treatment enabled neurons to establish more effective long-term potentiation (LTP) was examined. Using brain slices from B6 control mice and APP/PS1 mice with and without CART treatment, it was found that the slope of the I/O curve obtained from APP/PS1 mice (B = 0.56; n = 12 slices/6 mice) was significantly less than in control B6 mice (B = 1.14; *P *< 0.01, n = 12 slices/6 mice) (Fig. 6a). APP/PS1 mice with CART treatment showed significantly improved slope of the I/O curves (B = 0.75; n = 12 slices/6 mice; p < 0.05 versus APP/PS1 mice without CART treatment), but still less than in B6 mice (Fig. 6a). In B6 mice, LTP was able to be stably induced (151.4 ± 7.15% at 55-60 min post-HFS; n = 12 slices/6 mice; Fig. 6b) by a high-frequency stimulus (HFS; 100 pulses at 100 Hz). However, the same HFS was unable to induce any increase in EPSP slope, with no LTP induction in APP/PS1 mice (100.43 ± 6.18%; n = 12 slices/6 mice; Fig. 6c). CART-treated APP/PS1 mice exhibited an increase in EPSP slope upon stimulation, resulting in the induction of LTP (129.35 ± 3.95%; n = 12 slices/6 mice; Fig. 6d).

### CART protects against mitochondrial dysfunction by neutralizing oxidative stress and mitochondrial DNA damage *in vitro*

**Figure 7:**
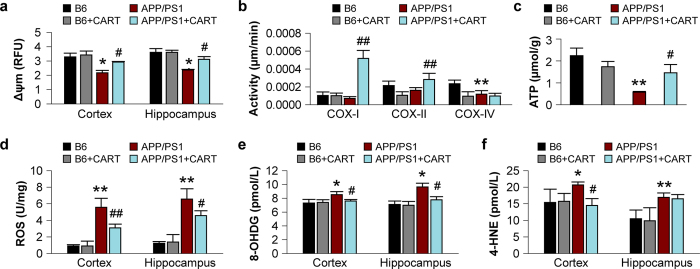
CART prevented mitochondrial dysfunction. (**a**) CART prevented the collapse of mitochondrial membrane potential (Δψm) in the hippocampus and cortex (increased by 31.1% and 35.0%). n = 3 mice/group. (**b**) CART stimulated mitochondrial complex-I and complex-II activities but not complex IV in the cortex of 8-month-old APP/PS1 mice. n = 3 mice/group. (**c**) CART attenuated the decrease in intracellular ATP levels in the cortex of APP/PS1 mice (increased by 140.3%). n = 3 mice/group. (**d**) CART attenuated intracellular ROS levels in the cortex (decreased by 44.6%) and hippocampus (decreased by 30.0%), as quantified by colorimetric spectrophotometry. n = 3 mice/group. (**e**) CART treatment reduced mitochondrial DNA damage, as reflected by a drop in 8-OHdG levels in the cortex (10.9%) and the hippocampus (19.2%). (**f**) CART treatment reduced lipid peroxidation, as indicated by 4-HNE levels (30.0%) in the cortex but not the hippocampus. All values are expressed as mean ± SEM from 3 mice in each group. *p < 0.05, **p < 0.01 versus B6 control mice, ^#^*p *< 0.05, ^##^*p *< 0.01 versus APP/PS1 mice without CART treatment.

We next examined the effects of CART treatment on the functional status of mitochondria in 8-month-old APP/PS1 mice compared to age-matched B6 control mice. APP/PS1 mice had substantially decreased membrane potential (34.1% in cortex and 34.0% in hippocampus) compared to B6 mice. CART treatment attenuated the reduction in mitochondrial membrane potential in APP/PS1 mice (improved by 35.0% in cortex, p < 0.05; and 31.1% in hippocampus, p < 0.05) (Fig. 7a), suggesting that CART ameliorated mitochondrial dysfunction. Furthermore, the activities of mitochondrial complex I and II, but not complex IV, were increased after CART treatment (Fig. 7b). In addition, neuronal ATP levels in APP/PS1 mice were ameliorated by CART, which elevated neuronal ATP by 140% (p < 0.05) compared to APP/PS1 mice without CART treatment (Fig. 7c).

In many neurological disorders, mitochondrial dysfunction is mainly due to excessive oxidative stress^14^,^15^. Levels of ROS, 8-OHdG and 4-HNE in the cortex and hippocampus of 8-month-old APP/PS1 transgenic mice and age-matched B6 control mice were compared, which indicated that ROS levels were significantly higher in APP/PS1 mice than in age-matched B6 mice (4.27-fold and 5.23-fold over B6 mice in the cortex and hippocampus, respectively, p < 0.01). In CART-treated APP/PS1 mice, ROS levels were reduced compared to levels in non-treated APP/PS1 mice (44.6% in the cortex, p < 0.01, and 30.3% in the hippocampus, p < 0.05, Fig. 7d). Similarly, compared to B6 control mice, APP/PS1 mice exhibited higher levels of 8-OHdG (an indicator of DNA damage from long-term exposure to excessive oxidative stress in the cortex and hippocampus) and CART treatment significantly reduced the level of 8-OHdG (10.9% in the cortex and 19.2% in the hippocampus, p < 0.05) (Fig. 7e). In addition, the level of 4-HNE was increased in APP/PS1 mice compared to that in B6 mice (25% in the cortex and 65% in the hippocampus, p < 0.05 and p < 0.01 respectively). CART treatment of APP/PS1 mice decreased the level of 4-HNE in the cortex by 30% (p < 0.05), but not in the hippocampus (Fig. 7f). Subsequent comparisons of 3-nitrotyrosine levels showed no significant differences between APP/PS1 and B6 mice (data not shown), suggesting that nitrative stress did not play a prominent role in the pathology of APP/PS1 mice.

## Discussion

This study demonstrates that CART is localized in Aβ plaques in the cortex and hippocampus of APP/PS1 mice and in AD brains and that pathological changes in APP/PS1 mice can be significantly ameliorated by CART treatment. CART is a well-characterized neuropeptide with the capacity to modulate body weight and alcohol consumption^16^,^17^. Recently, protection against cellular degeneration, which is an alternative CART function, has received growing attention, although studies in this area are still limited^2^,^3^,^4^,^7^,^9^. In a previous *in vitro* study by the current study team, it was discovered that upregulation of CART by icariin treatment reduced the death rate of cultured cortical neurons in response to Aβ toxicity^6^. The present study elucidates the effects of CART treatment on memory deficits and synaptic loss in APP/PS1 transgenic mice. The study found that CART treatment significantly attenuated memory deficits. Injections of exogenous CART re-established synaptic ultrastructure in the cortex and hippocampus, increased synapse numbers, and preserved LTP. CART also mitigated potential for depolarization of the mitochondrial membrane, a major cause of mitochondrial dysfunction, possibly by its ability to reduce intracellular ROS and lipid peroxidation and to decrease mitochondrial (mtDNA) oxidative damage.

In the brains of AD patients and 6, 8, and 10-month-old APP/PS1 mice, it was observed that CART immunoreaction surrounded and/or was in Aβ plaques. The pathogenesis of β-amyloid plaques may lie in the generation of dystrophic neurites as a result of mitochondrial collapse in response to ROS and lipid peroxidation^18^,^19^,^20^. In the current study, CART was shown to improve structure of neurites and reduce ROS, 8-OHdG and 4-HNE levels, two robust indicators of oxidative stress, and these changes were associated with improved mitochondrial function, consistent with published reports^7^,^21^. These results suggest that CART has the capacity to counter neuronal oxidative stress and mitochondrial dysfunction. Therefore, the accumulation of CART in Aβ plaques may serve as a compensatory mechanism to delay degenerative processes in dystrophic neurites and suppress Aβ plaque toxicity. Because neuritic plaques are a major histopathological hallmark of AD, the potential neuroprotective effects of CART against Aβ toxicity suggest that CART is a rational target in the search for therapies against this disorder. Furthermore, the mechanism underlying CART-mediated protection also warrants further investigation.

It has been consistently observed that significant degeneration of synaptic ultrastructure was correlated with an inability to induce LTP in 8-month-old APP/PS1 mice^22^. CART treatment of APP/PS1 mice boosted synaptophysin expression levels in the cortex and hippocampus, re-established synaptic ultrastructure, and increased LTP induction. These results suggest that CART promotes recovery of synaptic processes and may provide a novel strategy for functional recovery. In addition, CART has been shown to elevate mRNA levels of several genes related to synaptic plasticity and memory formation, such as Homer1a^23^,^24^, Arc^23^,^25^ and Nur77^26^,^27^ (data not shown). Further studies are needed to elucidate the relationships of these genes to CART expression in order to better understand the mechanism underlying CART protection *in vivo*.

In this study, a major target of CART protection was mitochondrion. The capacity of CART to lower oxidative damage of mtDNA in APP/PS1 mice is consistent with *in vitro* studies^7^. In addition, the observed reduction in ROS and 4-HNE in the presence of CART may also contribute to an attenuation of mitochondrial dysfunction by CART in ischemia and Parkinson’s disease models^7^,^21^. Mitochondrial fission and fusion have been shown to be compensatory responses to stress^14^,^28^,^29^. The current study showed that CART modulated the mRNA of genes related to fission and fusion. For example, Drp1 mRNA was markedly decreased and Fis1 mRNA was increased in APP/PS1 mice (Supplementary Fig. 1), suggesting that mitochondrial fission was stimulated. CART treatment reversed these changes in APP/PS1 mice and returned expression levels to that of B6 control mice. Whether these two genes mediate the effects of CART on the pathology of AD is currently being investigated.

In conclusion, this study demonstrates that CART has multiple protective effects in APP/PS1 mice, such as recovery of synaptic ultrastructure, amelioration of memory deficits, and protection of mitochondrial dysfunction from excessive oxidative stress and mtDNA damage. Further research on CART as a target for neuroprotection against AD pathology is warranted.

## Methods

### Animals and treatment

Male APP/PS1 transgenic mice^30^ were obtained from Nanjing University. Age-matched B6 control mice and APP/PS1 mice were housed in cages with a 12-hour light/dark cycle under controlled temperature (23 ± 2 °C) and humidity (50 ± 10%) conditions until 4, 6, 8, and 10 months of age. CART peptide was purchased from Phoenix Pharmaceuticals and dissolved in saline. At the age of 8 months, transgenic and control mice were randomly divided into CART-treated or normal saline-treated groups (15-20 mice per group). CART was injected via caudal vein daily at a dose of 0.5 μg/kg for 10 days, and then intraperitoneally injected daily for 20 days. All animals were handled according to the regulations of the Experimental Animal Administration issued by the State Committee of Science and Technology of the People’s Republic of China. All procedures were approved by Nanjing University’s Committee of Experimental Animal Administration.

### Cell culture and Aβ treatment

Primary cortical neurons were prepared from 16- to 17-day old mouse embryos, as described previously^6^. Briefly, dissociated cortical cells were plated on poly-D-lysine-coated plates at a density of 2 × 10^5^ cells/ml, and were maintained in Neurobasal media supplemented with B27 (Invitrogen, USA) and 25 nM glutamine. Synthetic Aβ_1-42_ (2 μM) was dissolved in 1% aqueous ammonia and added to primary neurons on day-*in-vitro* 8 (DIV8) for 24 hours. To determine the protective effects of CART, CART peptide (0.4 nM) or saline was applied on cultures 1 hour before Aβ_1-42_ treatment.

### AD patient and control brain samples

Cortical tissues from three AD patients and three age-matched non-demented individuals were obtained from the Alzheimer’s Disease Research Center (ADRC) brain bank, University of Pittsburgh.

### Immunostaining

Cortical neurons were fixed with cold acetone for 10 minutes followed by permeabilization with 0.1% Triton-X100 for 20 minutes at room temperature. The fixed and permeabilized neurons were then blocked with 5% goat serum in phosphate-buffered saline (PBS) with 0.05% Triton-X100 (PBST) for at least 1 hour at room temperature. Neurons were subsequently incubated with anti-MAP2 antibody (1:1000, Millipore) and anti-synaptophysin antibody (1:2000, BD Transduction Laboratories) overnight at 4 °C. After 3 consecutive 10-minute washes with PBST, neurons were incubated with the appropriate secondary antibody (Santa Cruz Biotechnology) for 2 to 4 hours at room temperature in the dark.

After 3 additional washes with PBST, samples were examined using confocal microscopy. The length of neurite positive for MAP2 and the area of positive puncta to synaptophysin were determined using NIH ImageJ software. Average neurite lengths (μm) were calculated as total neurite lengths of MAP2 positive cells divided by the total number of neurons within each image. Any puncta associated with a neurite or a cell body was involved in the analysis. Average area (μm^2^) of synaptophysin positive puncta on each neurite was measured as total puncta areas divided by the length of neurite. Each readout per well was averaged from images of 8 fields per well; an average of 60 to 80 cells were counted per well. The final quantification was based upon at least three independent experiments.

Control B6 and APP/PS1 transgenic mice (n = 3 per group) were anesthetized with 1% pentobarbital and sacrificed by intracardial perfusion with cold saline followed by 4% paraformaldehyde. The brain tissues were fixed in 4% paraformaldehyde for 24 hours, after which they were transferred into 15% and 30% sucrose in PBS overnight at 4 °C. Brains were harvested and sectioned (10 μm) in the coronal plane on a cryostat^31^. Each section was affixed on glass slides with 4% paraformaldehyde and washed with PBS. After blocking in 10% normal sera (Jackson), sections were incubated in antibodies against CART (1:2000; Phoenix Pharmaceuticals) and Aβ_1-42_ (clone: DE2B4, 1:200; Abcam) overnight at 4^°^C. Sections were then incubated with the appropriate secondary antibodies (avidin–biotin–HRP complex) for 2 hours at room temperature in the dark.

Tissue sections (40 μm) from cortex of AD patients and control individuals were also incubated in CART antibody (1:1000; Phoenix Pharmaceuticals) for 1 hour at room temperature, followed by appropriate biotinylated secondary antibodies for 2 hours at 37 °C, and by avidin-biotin complex reagents and peroxidase substrate. Images were captured using a Nikon TE200 microscope with a Spot RT digital camera and analyzed via Adobe Photoshop 5.5 software (Adobe Systems). Sections through the cortex and hippocampus were analyzed for Aβ plaque numbers and percent area covered by Aβ plaques with NIH ImageJ software using the ‘Analyze Particles’ function (5 brain sections per mouse). All measurements were made by blinded investigators.

### Exogenous CART distribution in mouse brain

Human CART peptide (Phoenix Pharmaceuticals) was radiolabeled with ^131^I using the chloramine-T method adapted from previously described techniques^32^. The specific incorporation of ^131^I into CART was over 90%. For each mouse, 200 μL of ^131^I-CART (equivalent to 40 μCi) was injected through the caudal vein, with ^131^I used as a control. At 10, 30, 60 and 120 minutes post-injection, mice were anesthetized with 1% sodium pentobarbital and sacrificed by intracardial perfusion with cold saline (n = 6 per group). The cortex, hippocampus, thalamus, striatum, cerebellum and pons were dissected from the brains and placed on ice. The CART radioactivity was determined for each brain region.

### Quantification of endogenous CART concentration in mouse brain

The mRNA level of endogenous CART was detected by real-time PCR as described previously^33^. Briefly, total RNA of the brain was isolated using the Trizol reagent (Invitrogen) and reverse transcription was performed using a PrimeScript RT reagent kit (Takara). The relative mRNA level was calculated after normalization to GAPDH mRNA. The primers used were as follows:

CART: Forward: 5′-GCCAAGTCCCCATGTGTGAC-3′, Reverse: 5′-CACCCCTTCACAAGCACTTCA -3′;

GAPDH: Forward: 5′-GCCAAGGCTGTGGGCAAGGT-3′, Reverse: 5′-TCTCCAGGCGGCACGTCAGA-3′.

The protein level of CART was determined using the CART ELISA kit (Ray Biotech, Norcross, GA, USA) according to the manufacturer’s instructions. Brain tissues were homogenized in cold PBS and quantified by BCA protein assay. The microplate in the kit was pre-coated with anti-rabbit secondary antibody. After incubation of the plate with rabbit anti-CART antibody, both biotinylated CART peptide and CART peptide in the homogenates interacted competitively with the CART antibody. Bound biotinylated CART peptide then interacted with Streptavidin-horseradish peroxidase (SA-HRP), which catalyzed a color development reaction. The intensity of colorimetric signal was directly proportional to the amount of biotinylated peptide-SA-HRP complex and inversely proportional to the amount of CART peptide in the homogenates of brain tissues (n = 6 per group).

### Morris water maze test

The Morris water maze was considered to be the most sensitive test of spatial memory and was performed as previously described^33^. The mice were blindly coded and the investigators were blinded to the groups during the test. Special attention was paid to avoid potential interferences that were manageable, such as room temperature, water temperature, lights and sound cues, and dimensions of the pool. To reduce the potential interferences, the injection and MWM tests were performed in different rooms by different investigators, and three days were allowed for all mice (n = 15 per group) to acclimate to the room where the maze was performed. During the training period, mice were allowed to locate a hidden platform (11 cm) which was submerged 1.5 cm below the water for 4 consecutive days. Each mouse was randomly released from one of 4 locations and had 60 seconds to search for the hidden platform. The mouse was guided to the platform for another 15 seconds if it could not locate the platform within 60 seconds. The investigator would sit behind the poster boards during each trial after releasing the mouse. Four trials per day for 4 consecutive days were conducted. A video camera was installed above the pool to track the mice. The time spent finding and mounting the platform (escape latency) and swimming speed were recorded using MWM software (Shanghai Yishu Software Technology Co. Ltd., China). After the last day of the hidden platform test, a single, 60-second probe-trial was performed with the platform removed. The numbers of passes over the previous location of the platform was recorded every 60 seconds. At the same time, the time spent in the target quadrant (where the platform was previously located) and swimming speed were recorded.

### Electrophysiology

All mice (n = 6 per group) were decapitated under deep anesthesia with ethyl ether. The brains were rapidly removed and coronal brain slices (400 μm) were cut using a vibrating microtome (Microslicer DTK 1500, Dousaka EM Co, Kyoto, Japan) in ice-cold cutting solution composed of the following: sucrose 94 mM, NaCl 30 mM, KCl 4.5 mM, MgCl_2_ 1.0 mM, NaHCO_3_ 26 mM, NaH_2_PO_4_ 1.2 mM, D-glucose 10 mM, and pH 7.4. The hippocampal slices were continuously perfused with artificial cerebrospinal fluid (ACSF) at 36 ± 1 °C for more than 60 minutes for recovery. ACSF was composed of the following: NaCl 124 mM, CaCl_2_ 2.0 mM, KCl 4.5 mM, MgCl_2_ 1.0 mM, NaHCO_3_ 26 mM, NaH_2_PO_4_ 1.2 mM, D-glucose 10 mM, and pH 7.4. Both the cutting solution and ACSF were oxygenated with a gas mixture of 95% O_2_/5% CO_2_. Slices were then transferred to a recording chamber and perfused with oxygenated ACSF (1-2 mL/min).

Orthodromic stimuli were delivered using an electrically polished bipolar tungsten electrode to stimulate the Schaffer collateral/commissural pathway. Constant current pulses (0.1 ms, 0.05 Hz) were supplied by a stimulator (SEN-3301, Nihon Kohden, Japan). Excitatory postsynaptic potentials (EPSPs) were recorded from the radiatum layer of the CA1 region and connected to a preamplifier with a high-pass filter at 5 kHz. Signals were amplified using a differential AC amplifier (A-M Systems, model 1700, Seattle, WA). EPSPs were digitized with the pCLAMP system (Axon Instrument Inc., Foster City, CA). Input/output (I/O) function was measured by averaging the slope of EPSPs against the stimulus intensity at 0.1-1.1 mA. Long-term potentiation (LTP) was generated by high-frequency stimulus (HFS, 100 pulses at 100 Hz) at the same intensity as pre-HFS. Subsequently, single pulse recording was resumed and continued for 60 min. The EPSP slope, when increased 20% above baseline continuously for over 60 minutes, was considered to be LTP induction.

### TEM Analysis

Mice were perfused with 0.9% saline under deep anesthesia, followed by a mixture of 4% paraformaldehyde and 2% glutaraldehyde (n = 3 per group). Perfused mice were then decapitated and the brains rapidly removed. The frontal cortex and the CA1 region of the hippocampus were cut and reframed into 1 mm^3^ blocks which were then fixed overnight in 2.5% glutaraldehyde solution. Subsequently, the blocks were fixed with 1% osmium tetroxide and immersed in Epon 812 resin/acetone (1:1) for 30 minutes, and then embedded in epoxy resin and incubated at 37 °C for 24 hours and 60 °C for 48 hours. Blocks were then cut into 50-nm sections and stained with 2% uranyl acetate and lead citrate for ultrastructural examination. The ultrastructure was observed using H-7000FA transmission electron microscopy (TEM) (Hitachi Co., Ltd., Tokyo, Japan). Photos were taken at 5,000 to 10,000 magnification (5 photos/section; 3 sections/mouse; 3 mice/group). A synapse was defined as having both a postsynaptic density and at least two vesicles in the presynaptic density^34^.

### Measurements of mitochondrial membrane potential

Changes in mitochondrial membrane potential (Δψ) of hippocampus and cortex (n = 3 per group) were measured as previously described^35^. The mitochondria of brains were isolated using the Tissue Mitochondria Isolation Kit (Beyotime, Nanjing, China). Hippocampus tissues were immediately removed and washed by phosphate buffered saline (PBS). Then the tissues were homogenized with ten times volume of isolation buffer on ice, and centrifuged at 600 g for 5 minutes at 4 °C. The supernatant was centrifuged again at 11, 000 g for 10 minutes at 4 °C to obtain the cytosol (supernatant) and mitochondria (deposition) fraction. The purified mitochondria and JC-1 staining solution (Beyotime, China) were mixed at 1:9. To evaluate the OD of JC-1, wavelengths of excitation and emission were set at 490 nm and 530 nm.

### Measurement of intracellular reactive oxygen species

For determination of intracellular reactive oxygen species (ROS) levels, samples of hippocampus and whole cortex from each group (n = 3 per group) were prepared according to the manufacturer’s instructions (Jianchen, Nanjing, China). After preparation, the samples were re-suspended in 10 mM 2, 7-dichlorodihydrofluorescein diacetate (DCFH-DA) solutions, followed by 20 minutes’ incubation at 37 °C. Then the samples were subjected to colorimetric spectrophotometry to measure intracellular ROS levels.

### Analysis of mitochondria complex activity and ATP levels

Activities of mitochondria complex I (NADH-ubiquinone oxidoreductase), complex II (succinate-ubiquinone oxidoreductase), complex III (ubiquinol-cytochrome c oxidoreductase), and complex IV (cytochrome c oxidase) were determined by colorimetric spectrophotometry (n = 3 mice per group) using commercially available kits (Genmed Scientifics Inc., USA). Briefly, mitochondrial suspension was incubated with the corresponding substrate (NADH, succinate and ferrocytochrome c ) at 25 °C for 1 minute, and the change in absorbance at 340 nm, 600 nm and 550 nm was measured using the spectrophotometer. Background levels were measured in the absence of mitochondrial suspensions and activity was expressed as micromoles of substrate oxidized per min–1 mg–1 protein. The ATP level was detected using the Enhanced ATP Assay Kit (Beyotime, Nanjing, China). The isolated mitochondria (n = 3 per group) were lysed in an equal volume of lysis buffer, and the content of ATP was measured by comparing the relative luciferase unit (RLU) of the sample to the standard curve of known ATP concentrations.

### Measurement of 4-HNE and 8-OHdG

Intracellular 4-hydroxy-2-nonenal (4-HNE) and 8-hydroxydeoxyguanosine (8-OHdG) levels in the hippocampus and cortex (n = 3 per group) were measured as previously described^36^. Briefly, the samples were added into the microplate pre-coated with mouse 4-HNE or 8-OHdG antibody. After incubation with 4-HNE or 8-OHdG antibody with labeled HRP at 4 °C overnight, TMB substrate solution and stop solution was then added. The absorbance was measured at a wavelength of 450 nm, and the concentration of 4-HNE or 8-OHdG was determined by comparing the O.D. of the sample to the standard curve.

### Western blot

Western blots were performed according to previously published techniques^4^. Equal amounts of total protein from each hippocampal sample (n = 3 per group) were resolved by SDS-PAGE and transferred onto PVDF membranes. Membranes were probed with primary antibodies recognizing synaptophysin (1:5000, BD Transduction Laboratories; 611800). The relative amount of synaptophysin in each sample was detected using a chemiluminescence ECL kit (Amersham Pharmacia Biotech, Piscataway, NJ, USA). Relative intensity of each band was measured using NIH ImageJ software. All experiments were performed at least in triplicate.

### Statistical analysis

Data were presented as mean ± SEM. Differences between groups were analyzed with a *t-test* for two groups and one-way ANOVA with *post hoc* tests for multiple groups (SPSS 16.0 system, SPSS, Chicago, USA). The criterion for statistical significance was set at *p* < 0.05.

## Author Contributions

Y.X. designed the experiments, wrote and edited the manuscript. A.L. interpreted the results, formulated and optimized the logic flow, and wrote the manuscript. Y.S. and J.C. interpreted the results, wrote and edited the manuscript. J.J., K.Y., X.Z. and L.L. performed the staining, cell culture and molecular biological experiments. L.L. performed the MWM tests, cell culture and molecular biological experiments. L.Q. performed the statistical analysis. L.C. and R.Y. performed the electrophysiological analysis. All authors reviewed the manuscript.

## Additional Information

**How to cite this article**: Jin, J.-L. *et al.* CART treatment improves memory and synaptic structure in APP/PS1 mice. *Sci. Rep.* **5**, 10224; doi: 10.1038/srep10224 (2015).

## Supplementary Material

Supplementary Information
