# Supplementary material for: CART treatment improves memory and synaptic structure in APP/PS1 mice
Source: Sci Rep. 2015 May 11;5:10224. doi: 10.1038/srep10224 (PMC4426675; doi:10.1038/srep10224)
Supplement: Supplementary Information [file srep10224-s1.pdf]

CART treatment improves memory and synaptic structure in APP/PS1 mice

Jia-li Jin, Anthony K.F. Liou, Yejie Shi, Kai-lin Yin, Ling Chen, Ling-ling Li, Xiao-lei Zhu, Lai Qian, Rong Yang, Jun Chen, and Yun Xu

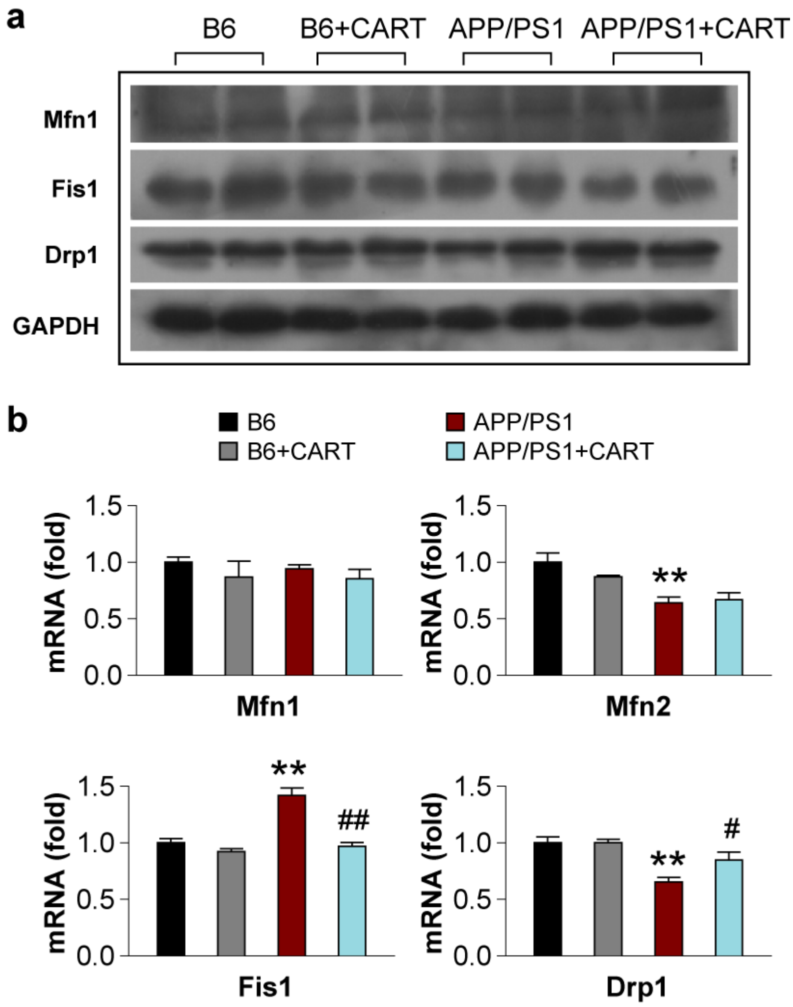

Supplementary Figure 1: CART treatment modulates the mRNA levels of mitochondrial fission/fusion genes. (a) CART treatment did not affect the protein levels of Mfn1, Fis1, and Drp1 in the hippocampus of 8-month-old APP/PS1 mice. The blot shown here is a representative of 3 independent experiments. Blot images were cropped for comparison. (b) CART treatment significantly increased the mRNA level of Drp1, and decreased the mRNA level of Fis1 in the hippocampus of APP/PS1 mice. $^{**}p<0.01$ versus B6 control mice, $^{\#}p<0.05$, $^{\#\#}p<0.01$ versus APP/PS1 mice without CART treatment.
